# High-Accuracy Calibration Method of a Thermal Camera Using Two Reference Blackbodies

**DOI:** 10.3390/s24175831

**Published:** 2024-09-08

**Authors:** Tomasz Sosnowski, Mariusz Kastek, Krzysztof Sawicki, Andrzej Ligienza, Sławomir Gogler, Bogusław Więcek

**Affiliations:** 1 Institute of Optoelectronics, Military University of Technology, 00-908 Warsaw, Poland; mariusz.kastek@wat.edu.pl (M.K.); krzysztof.sawicki@wat.edu.pl (K.S.); andrzej.ligienza@wat.edu.pl (A.L.); slawomir.gogler@wat.edu.pl (S.G.); 2 Institute of Electronics, Lodz University of Technology, 90-924 Łódź, Poland; boguslaw.wiecek@p.lodz.pl

**Keywords:** non-contact temperature measurement, thermal camera model, signal procesing

## Abstract

Body temperature is one of the most important physiological parameters of a human being used to assess his basic vital functions. In medical practice, various types of measuring instruments are used to measure temperature, such as liquid thermometers, electronic thermometers, non-contact ear thermometers, and non-contact forehead thermometers. Such body temperature measurement techniques require the connection of appropriate sensors to a person, and non-contact thermometers operate over short distances and force a specific position of the person during the measurement. As a result, using the above methods, it is practically impossible to perform body temperature measurements of a moving human being. A thermal imaging camera can be used effectively for the purpose of the temperature measurement of moving objects, but the remote measurement of a human body temperature using a thermal imaging camera is affected by many factors that are difficult to control. Accurate remote measurement of human body temperature requires a measurement system that implements a specialized temperature determination algorithm. This article presents a model of a measurement system that facilitates the development of a highly accurate temperature measurement method. For the model, its parameters were determined on the calibration stand. The correct operation of the developed method and the effectiveness of temperature measurement have been confirmed by tests on a test stand using reference radiation sources.

## 1. Introduction

Technological developments in the field of infrared and thermal imaging have resulted in thermal imaging cameras being used in a wide range of applications in areas such as military [1], police equipment, medicine, scientific research, as well as [2] automotive, alarm systems, and production surveillance systems. One common medical application of a thermal imaging camera is the measurement of human body temperature.

Measurement and monitoring of human physiological parameters play an important role in many applications such as healthcare, psycho-physiological research (e.g., polygraph), sports training, and research on the effectiveness of therapies and the spread of diseases. Dynamic changes in physiological parameters can reveal changes in a patient’s physiological state and function. In addition to health status, they can be used to assess a person’s activity status, performance, and fatigue. Among the most important physiological parameters for assessing a person’s basic vital functions, alongside heart rate, blood pressure, and respiratory rate, is body temperature.

Due to the fact that heat is generated and absorbed by all tissues and organs of the human body, the value of the internal temperature of the human body is characterized by a non-uniform spatial distribution. In general, internal body temperature refers to the temperature of the organs responsible for blood distribution in the brain, chest, and abdominal cavity. Accurate measurement of internal temperature is only possible using invasive methods, by placing the measuring probe in internal organs such as the oesophagus, pulmonary artery, and urinary bladder. However, it is difficult and impractical to carry out temperature measurements in internal organs. In addition, high accuracy of internal temperature measurement is necessary only for critically ill patients. In many cases, temperature measurement can be carried out non-invasively, at more easily accessible sites whose temperature closely matches the internal body temperature. Such sites include the anus, oral cavity, axillary fossa, temporal artery (on the forehead or the temple), and external auditory canal.

In medical practice, different types of temperature-measuring instruments are used, such as liquid thermometers, electronic thermometers, non-contact ear thermometers, and non-contact forehead thermometers. Some of the above techniques for measuring body temperature require attaching appropriate sensors to a person. However, it may be undesirable or impossible to attach the sensors to a person, such as in newborns, during sports training, or when examining large numbers of moving people. Non-contact thermometers operate at short distances and often force a certain position of the person during the measurement. In addition, increasing the distance makes it difficult to hold the measuring point at the required location like the forehead, temple, or ear. This makes it virtually impossible to utilize such methods while a person is moving.

Measuring the temperature of moving objects with a thermal imaging camera can be effective; however, it involves complex considerations of often non-obvious parameters specific to a particular situation, making it a challenging process. Despite the fact that the design and software of today’s measurement thermal imaging cameras has made it much easier to use them, in order to obtain accurate, reliable results, the user must know and understand the basics of thermography, infrared radiation propagation, optical systems, and calibration methods. Often, the user also needs to have considerable experience in thermal imaging measurements. In addition, thermal cameras measure absolute temperature with an accuracy of ±2 °C. The aforementioned considerations for thermal imaging measurements can lead to significant errors in measuring human body temperature.

## 2. Composition of the Measurement System

In the developed measurement system, remote temperature measurement with a thermal imaging camera is achieved by recording the distribution of infrared radiation emitted from the skin of observed people. The measurement of human skin temperature is affected by many factors. The camera’s software calculates the skin temperature distribution of observed individuals by considering factors such as the known emissivity of human skin, ambient temperature, calibration data, and parameters like transmission and temperature of the optical system. Additionally, it takes into account external temperature, camera temperature, and detector temperature while considering its self-heating. To enhance temperature measurement accuracy, a set of temperature standards has been placed in the field of view of the thermal imaging camera. The set of temperature standards used in the system is composed of two blackbodies (BBs) with high temperature stability, constructed and fabricated in the Institute of Optoelectronics, Military University of Technology (IOE MUT) equipped with temperature stabilization circuits and communication and control interfaces.

Thus, achieving effective remote measurement of human skin temperature requires a thermal imaging camera equipped with the following elements:

- thermal camera module (IR camera module),
- shutter module,
- VIS camera module (visible range camera),
- control and data processing module,
- a set of temperature standards.

**Figure 1:**
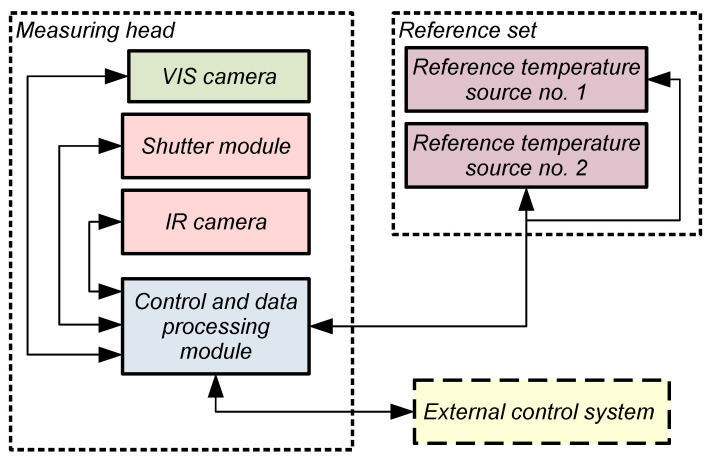
Block diagram of an assembly for remote temperature measurement using a thermal imaging camera.

Figure 1 shows a general block diagram of an assembly for remote measurement of human skin temperature using a thermal imaging camera.

One of the most important activities carried out in the remote temperature measurement assembly is auto-calibration, which allows the data processing parameters to be set for the current state of the camera. For this purpose, a shutter was used to capture a reference image. A set of additional thermal sensors was used for recording data such as internal temperature and aperture’s temperature. Based on this information, the thermal camera calculates the coefficients and parameters.

Based on the collected data, the thermal imaging camera determines radiometric values for each sensor of the array depending on the incident radiation and compiles them into a data table, which is then sent to the control and data processing module. The control and data processing module determines temperature values by performing calculations based on the data from the corresponding pixel, along with a set of coefficients and parameters.

**Figure 2:**
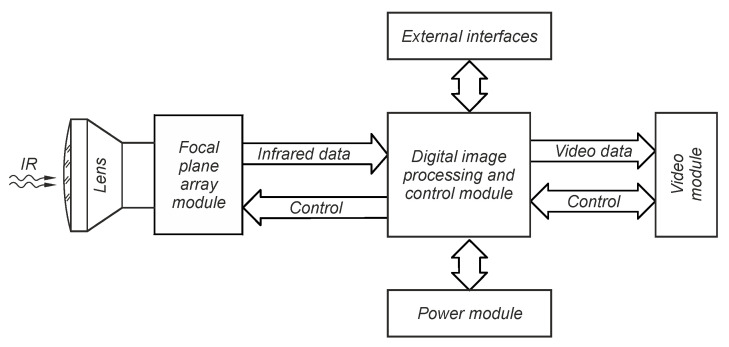
General concept of a detector module broken down into basic functional systems.

A measurement thermal imaging camera consists of the following components: an infrared detector array, the lens for a given infrared range, electronic circuits that provide detector signal reading, recording, and analysis, and a display module [2,3,4]. A general schematic of the IR detection module is shown in Figure 2.

The most important component of the thermal imaging camera is the infrared detector array. The system uses Lynred’s PICO640S microbolometer thermal image sensor with a resolution of 640 × 480 and a single detector (pixel) size of 17 µm. It is an uncooled detector array, capable of operating at room temperature. Bolometers work on the principle of changing the electrical resistance of the detector under the influence of a temperature change caused by absorption of incident radiation [5,6]. Based on the change in resistance, the readout integrated circuit (ROIC) generates a voltage signal corresponding to the change in energy of incident radiation absorbed by the detector.

The detector array is characterized by some inhomogeneity in the response of individual detectors excited by the same incident infrared radiation. This inhomogeneity leads to the occurrence of fixed pattern noise (FPN) in the thermal image generated by the detector array, stemming from technological variations in array parameters. Typically, the readout circuit integrated into the detector array does not implement the non-uniformity correction (NUC) procedure, which is instead performed by an external circuit. The most common methods of non-uniformity correction are single-point correction and two-point correction [7,8,9].

In the developed thermal imaging camera, the primary method of inhomogeneity correction is the two-point method, for which the correction coefficients were determined on a laboratory calibration bench. Additionally, to enhance correction quality, a single-point correction is performed using a shutter module with controlled temperature [9,10].

There are defects in each infrared focal plane array (FPA) that cause the array to have a certain number of faulty detectors commonly called “bad pixels” [8].

In the developed thermal imaging camera, inhomogeneity correction and replacement of defective pixels are carried out on an ongoing basis. The temperature determination algorithm performs calculations for the image after inhomogeneity correction and removal (replacement) of defective pixels.

Another important component of a measurement thermal imaging camera is the lens. The camera’s field of view (FOV) and instantaneous field of view (IFOV) [8] are determined from the parameters of the lens used. These parameters influence the number of pixels of the thermal image that make up the image of the face from a given distance. This parameter is very important in the context of measuring human body temperature. A sufficient number of image pixels allows more effective detection of a person’s face [11,12]. Moreover, measuring body temperature based on skin temperature in specific facial areas is more reliable. Increasing the field of view angle or the distance from the person being monitored reduces the number of imaging pixels capturing the face, thereby decreasing temperature measurement accuracy. For this reason, it is necessary to use a lens adapted to the measurement situation.

To ensure the optical and mechanical conditions necessary for accurate measurement of human skin temperature, a lens with a focal length of 19 mm was employed. The basic parameters of the lens are listed in Table 1.

## 3. Measurement System Model

### 3.1. Radiation Temperature Measurement Model

The thermal imaging camera receives not only infrared radiation from the observed object but also radiation from ambient elements (people, trees, buildings) and radiation reflected from the surface of the object. The camera also receives solar radiation, especially reflected radiation from the object and surrounding elements. All radiation components are attenuated by the atmosphere located in the path of radiation propagation. In order to obtain accurate temperature measurement results with a thermal imaging camera, it is necessary to consider the influence of various phenomena and radiation sources that may interfere with the measurement. Since estimating the mentioned interferences is challenging, mathematical models for temperature determination based on analysis of recorded infrared radiation are simplified to achieve the necessary accuracy while minimizing the number of analyzed parameters. Partial interference compensation can be performed based on data such as object emissivity, ambient temperature, distance between the object and the camera, relative humidity, temperature, and atmospheric transmission, in addition to the influence of temperature measurement of such factors as the temperature of the optical elements of the lens (including optical windows), the transmission coefficient of the optical elements, the temperature and transmission of the atmosphere, and the internal temperature of the camera, etc. Taking all phenomena into account at the highest level is most often impractical or prohibitively expensive. Therefore, trade-offs between quality and development and production costs are commonly made, which is typically reflected in the parameter values of the camera.

**Figure 3:**
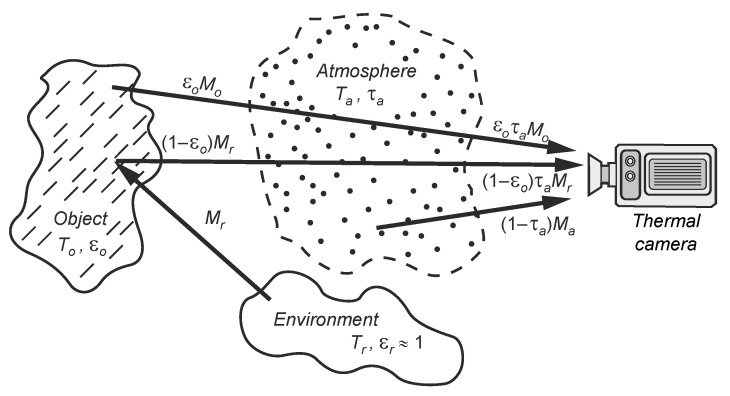
Infrared radiation model of the scene in thermographic measurements.

A thermal imaging camera receives radiation from many sources. Including all of them (interfering sources) in the temperature determination algorithm is impossible and often pointless. It is assumed that infrared radiation from the sources shown in Figure 3 reaches the camera.

According to the situation shown in Figure 3, the total radiation power received by the thermal imaging camera from the observed scene (reaching the camera lens) is:

(1) $$M_{C\lambda }=\varepsilon_{o\lambda }\tau_{a\lambda }M_{\lambda }\left(T_{o}\right)+\left(1-\varepsilon_{o\lambda }\right)\tau_{a\lambda }M_{\lambda }\left(T_{r}\right)+\left(1-\tau_{a\lambda }\right)M_{\lambda }\left(T_{a}\right),$$

where $M_{\lambda }\left(T\right)$ is the spectral exitance of a blackbody as a function of temperature *T*, $\varepsilon_{o\lambda }$ is the emissivity of the object, $\tau_{a\lambda }$ is the transmission coefficient of the atmosphere, $T_{o}$ is the temperature of the object, $\rho_{o\lambda }$ is the reflection coefficient which for non-transparent bodies is equal to $\rho_{o\lambda }\;=\;\left(1-\varepsilon_{o\lambda }\right)$, $\left(1-\tau_{a\lambda }\right)$ is the emissivity of the atmosphere (assuming $\rho_{a\lambda }=0$), and $T_{a}$ is the temperature of the atmosphere. The temperature of the sources in the environment is $T_{r}$, and we treat the entire environment as a blackbody with the same temperature.

**Figure 4:**
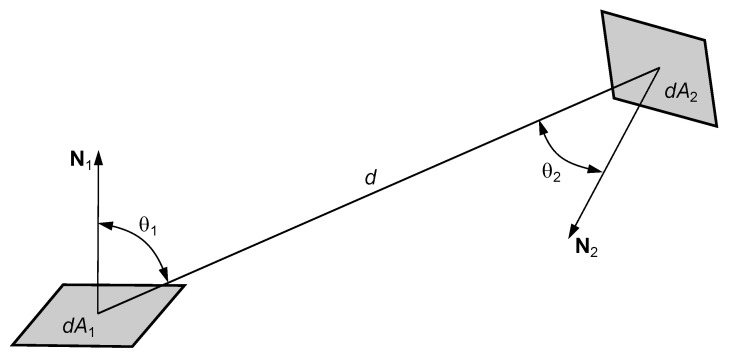
Geometric relationships for the radiation transfer between two surfaces $dA_{1}$ and $dA_{2}$.

Many parameters of thermal cameras influence the quality of performance and the accuracy of temperature measurement [7,12,13,14,15,16,17]. Therefore, a large number of physical phenomena such as power source noise, detector circuit noise, detector temperature, ambient temperature, digital data processing speed and method, and analog-to-digital processing parameters (e.g., quantization noise) must be taken into account when determining the temperature from radiation measurements with a thermal camera. The main parameter that has the greatest impact on the operation of the camera, especially with an uncooled detector array, is the internal temperature of the camera and its components, sensed by the detector. Within a thermal camera, there is an exchange of energy between the detector and all surrounding surfaces—a transfer of radiant power according to the fundamental law of radiometry [8,18,19,20,21,22,23,24]:

(2) $$d^{2}\Phi =\frac{L_{1}\cdot cos\theta_{1}\cdot cos\theta_{2}}{d^{2}}{dA}_{1}{dA}_{2},$$

where $L_{1}$ is the radiance of the surface $dA_{1}$. The radiant power emitted by surface $dA_{1}$ and received by surface $dA_{2}$ depends on the distance and relative orientation of the two areas relative to the connecting line. Figure 4 shows the geometric relationships for the radiative transfer between the two surfaces $dA_{1}$ and $dA_{2}$.

The above phenomenon was taken into account in the model by means of a configuration factor (view factor) [21,25] determined for each case considered, expressed by the general formula:

(3) $$F_{1-2}=\frac{\Phi_{1-2}}{\Phi_{1}},$$

where $\Phi_{1-2}$—radiation power radiated from surface 1 and incident on surface 2, $\Phi_{1}$—total radiation power emitted by surface 1 into half-space.

If the energy exchange occurs between surfaces $A_{1}$ and $A_{2}$ of finite dimensions, then the configuration factor can be expressed by the formula [8,22]:

(4) $$F_{1-2}=\frac{1}{\pi A_{1}}\int \;\;\;\int_{A_{1},A_{2}}\frac{cos\theta_{1}\cdot cos\theta_{2}}{d^{2}}{dA}_{1}{dA}_{2}.$$

Formula (4) does not cover all possible cases. However, it already shows that the analytical determination of the configuration factor can be very difficult even for surfaces with not very complicated shapes [21,25]. Therefore, numerically determined configuration factors were used in the model.

### 3.2. Radiation Model of the Camera

In order to determine the effect of radiation of the camera elements, a thermal camera model was developed taking into account the essential sources of radiation and the camera parameters affecting the measurement accuracy. In the developed thermal imaging camera model, it was assumed that the main elements of the camera are:

- *D* infrared detectors, each of which has an area $A_{D}$,
- the surface area of the detector housing *S*,
- the surface of the thermal camera housing *P* (the surface of the interior of the camera, ”seen” by the infrared detector),
- input window of the detector array (optical window) *W*,
- surface of the lens (detector side) *Q*,
- area of the interior of the lens *V*.

**Figure 5:**
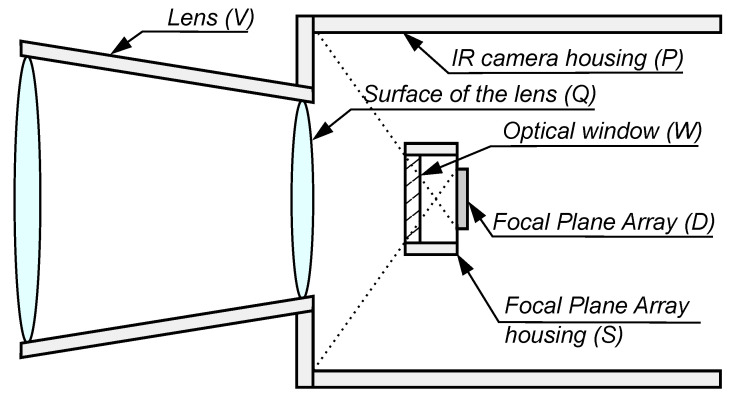
Schematic of the thermal imaging camera model.

The model allows estimating the amount of radiation power emitted by the above surfaces and incident on each detector of the array. In this manner, the model assumes that all radiating surfaces are Lambertian surfaces coated with paint, resulting in an emissivity greater than 0.96 (the cameras tested had surfaces made in this way). Thus, to simplify the model, only the emissivity of the radiation from these surfaces was considered, neglecting the reflection. Furthermore, anti-reflective layers were applied to the lens, which permitted the effect of reflection from the lens surfaces to be neglected in the model. Figure 5 shows a schematic of the detector array camera model.

For the assumptions above and using the reciprocity principle for the configuration coefficients, the power emitted by the model surfaces incident on the detector can be represented by the formulas:

(5) $$\Phi_{Q-dD}=A_{Q}F_{Q-dD}M_{Q}\left(T_{Q}\right)=A_{D}F_{dD-Q}M_{Q}\left(T_{Q}\right),$$

(6) $$\Phi_{V-dD}=A_{V}F_{V-dD}M_{V}\left(T_{V}\right)=A_{D}F_{dD-V}M_{V}\left(T_{V}\right),$$

(7) $$\Phi_{S-dD}=A_{S}F_{S-dD}M_{S}\left(T_{S}\right)=A_{D}F_{dD-S}M_{S}\left(T_{S}\right),$$

(8) $$\Phi_{W-dD}=A_{W}F_{W-dD}M_{W}\left(T_{W}\right)=A_{D}F_{dD-W}M_{W}\left(T_{W}\right),$$

(9) $$\Phi_{P-dD}=A_{L}F_{P-dD}M_{P}\left(T_{P}\right)=A_{D}F_{dD-P}M_{P}\left(T_{P}\right),$$

(10) $$\Phi_{tot}=\Phi_{Q-dD}+\Phi_{V-dD}+\Phi_{S-dD}+\Phi_{W-dD}+\Phi_{P-dD},$$

where $A_{D}$ is the area of the detector surface of the array, $\Phi_{X-dD}$—the power of radiation radiated from the surface of *X* and incident on the detector surface dD, $F_{X-dD}$—the configuration factor determining how much of the radiation power emitted from the surface of *X* reaches the detector surface dD, $A_{X}$—the area of the surface of *X*, $M_{X}$—the radiant exitance of the surface of *X*, $T_{X}$—the temperature of the surface of *X*, *X*—stands for the surface *Q*, *V*, *S*, *W*, *P*, respectively.

In developing the radiation model of the camera, it was postulated that a simplified model comprising a limited number of surfaces and parameters would serve to reduce the temperature measurement error. This approach enabled the calculations to be streamlined and the number of sensors installed in the camera to be reduced.

In the adopted camera model, it is assumed that the radiating surfaces are characterized by emissivity $\varepsilon_{X\lambda }$. Through the lens *Q*, each detector of the matrix receives radiation from the observed scene, defined by the radiant exitance values $M_{C\lambda }$, and radiation from the interior of the lens, defined by the exitance values $M_{V}$, and the lens is characterized by the transmission $\tau_{Q\lambda }$ and the transmission of the back lens $\tau_{Q\lambda }^{*}$. Then, the radiant exitance values of the model are defined by the formulas:

$$M_{Q}=\tau_{Q\lambda }M_{C\lambda }.$$

$$M_{V}=\tau_{Q\lambda }^{*}\varepsilon_{V\lambda }M_{\lambda }\left(T_{V}\right).$$

$$M_{X}\left(T\right)=\varepsilon_{X\lambda }M_{\lambda }\left(T\right),$$

where $M_{\lambda }\left(T\right)$ is the spectral exitance of a blackbody as a function of temperature *T*. In addition, the input window of the detector has a transmission $\tau_{W\lambda }$, and its emissivity is $\varepsilon_{W\lambda }=1-\tau_{W\lambda }$. In this case, the Formulas (5)–(9) take the form:

(11) $$\begin{array}{ccc} \Phi_{Q-dD} & = & A_{D}F_{dD-Q}\cdot \tau_{Q\lambda }\tau_{W\lambda }[\varepsilon_{o\lambda }\tau_{a\lambda }M_{\lambda }\left(T_{o}\right)+\\ & & \left(1-\varepsilon_{o\lambda }\right)\tau_{a\lambda }M_{\lambda }\left(T_{r}\right)+\left(1-\tau_{a\lambda }\right)M_{\lambda }\left(T_{a}\right)] \end{array}$$

(12) $$\Phi_{V-dD}=A_{D}F_{dD-V}\cdot \tau_{W\lambda }\tau_{Q\lambda }^{*}\varepsilon_{V\lambda }M_{\lambda }\left(T_{V}\right),$$

(13) $$\Phi_{W-dD}=A_{D}F_{dD-W}\cdot \varepsilon_{W\lambda }M_{\lambda }\left(T_{W}\right),$$

(14) $$\Phi_{S-dD}=A_{D}F_{dD-S}\cdot \varepsilon_{S\lambda }M_{\lambda }\left(T_{S}\right),$$

(15) $$\Phi_{P-dD}=A_{D}F_{dD-P}\cdot \tau_{W\lambda }\varepsilon_{P\lambda }M_{\lambda }\left(T_{P}\right).$$

The infrared array detector receives radiation in the band $\lambda \;\in \;\langle \lambda_{1},\lambda_{2}\rangle$ and is characterized by a voltage sensitivity $R_{v\lambda }$ defined by the formula [23,24]:

$$R_{v\lambda }=\frac{U_{\lambda }}{\Phi_{e\lambda }},$$

where $U_{\lambda }$ is the voltage signal induced by the radiation flux $\Phi_{e\lambda }$. If we additionally assume that for this wavelength range the values of all emissivities and transmission values are constant:

$$\varepsilon_{o\lambda }=\varepsilon_{o}=const,\;\varepsilon_{W\lambda }=\varepsilon_{W}=const,\;\varepsilon_{S\lambda }=\varepsilon_{S}=const,\;\varepsilon_{P\lambda }=\varepsilon_{P}=const,$$

$$\varepsilon_{V\lambda }=\varepsilon_{V}=const,\;\tau_{a\lambda }=\tau_{a}=const,\;\tau_{W\lambda }=\tau_{W}=const,\;\tau_{Q\lambda }=\tau_{Q\lambda }^{*}=\tau_{Q}=const,$$

then Formulas (11)–(15) can be written as follows:

(16) $$\begin{array}{ccc} U_{Q} & = & A_{D}F_{dD-Q}\cdot \tau_{Q}\tau_{W}\cdot \left[\varepsilon_{o}\tau_{a}\int_{\lambda_{1}}^{\lambda_{2}}R_{v\lambda }M_{\lambda }\left(T_{o}\right)d\lambda +\right.\\ & & \left(1-\varepsilon_{o}\right)\tau_{a}\int_{\lambda_{1}}^{\lambda_{2}}R_{v\lambda }M_{\lambda }\left(T_{r}\right)d\lambda +\left.\left(1-\tau_{a}\right)\int_{\lambda_{1}}^{\lambda_{2}}R_{v\lambda }M_{\lambda }\left(T_{a}\right)d\lambda \right], \end{array}$$

(17) $$U_{V}=A_{D}F_{dD-V}\cdot \tau_{Q}\tau_{W}\varepsilon_{V}\int_{\lambda_{1}}^{\lambda_{2}}R_{v\lambda }M_{\lambda }\left(T_{V}\right),$$

(18) $$U_{W}=A_{D}F_{dD-W}\cdot \varepsilon_{W}\int_{\lambda_{1}}^{\lambda_{2}}R_{v\lambda }M_{\lambda }\left(T_{W}\right)d\lambda ,$$

(19) $$U_{S}=A_{D}F_{dD-S}\cdot \varepsilon_{S}\int_{\lambda_{1}}^{\lambda_{2}}R_{v\lambda }M_{\lambda }\left(T_{S}\right)d\lambda ,$$

(20) $$U_{P}=A_{D}F_{dD-P}\cdot \tau_{W}\varepsilon_{P}\int_{\lambda_{1}}^{\lambda_{2}}R_{v\lambda }M_{\lambda }\left(T_{P}\right)d\lambda .$$

Taking the designation:

(21) $$U_{D}\left(T\right)=\int_{\lambda_{1}}^{\lambda_{2}}R_{v\lambda }M_{\lambda }\left(T\right)d\lambda ,$$

and assuming that the transmission of the input window of the detector is $\tau_{W}\approx 1$ ($\varepsilon_{W}\approx 0$) and in addition:

(22) $$\varepsilon_{S}=\varepsilon_{P}=\varepsilon_{H},\;T_{S}=T_{P}=T_{H},\;F_{dD-H}=F_{dD-S}+F_{dD-P},\;U_{H}=U_{S}+U_{P}=A_{D}F_{dD-H}\cdot \varepsilon_{H}U_{D}\left(T_{H}\right),$$

then we obtain the formula for the total voltage on the detector resulting from the incident radiation:

(23) $$\begin{array}{ccc} U_{tot} & = & A_{D}F_{dD-Q}\cdot \tau_{Q}\varepsilon_{o}\tau_{a}U_{D}\left(T_{o}\right)+\\ & & A_{D}F_{dD-Q}\cdot \tau_{Q}\left(1-\varepsilon_{o}\right)\tau_{a}U_{D}\left(T_{r}\right)+\\ & & A_{D}F_{dD-Q}\cdot \tau_{Q}\left(1-\tau_{a}\right)U_{D}\left(T_{a}\right)+\\ & & A_{D}F_{dD-V}\cdot \tau_{Q}\varepsilon_{V}U_{D}\left(T_{V}\right)+\\ & & A_{D}F_{dD-H}\cdot \varepsilon_{H}U_{D}\left(T_{H}\right). \end{array}$$

Determining by means of Formula (21), the value of the detector voltage $U_{D}$ as a function of the temperature *T* of the observed objects and for a limited spectral range ($\lambda \in \langle \lambda_{1},\lambda_{2}\rangle$) seems methodologically straightforward, especially when using numerical integration. However, this approach requires the knowledge of the detector’s spectral voltage sensitivity. Due to the significant variation in the fabrication of infrared detectors, it is necessary to measure this characteristic for each detector of the array, which is basically only possible at the detector manufacturing stage. Measuring the spectral sensitivity characteristics for each fabricated detector array is difficult and very expensive. Consequently, manufacturers provide sensitivity characteristics only for a family of detector arrays. These characteristics have limited spectral resolution and exhibit significant variability within the detector family.

Therefore, the best way to determine the detector voltage $U_{D}$ as a function of the temperature *T* of the observed objects for the spectral range is to use an approximating function according to the modified Sakuma–Hattori formula [26,27]:

(24) $$U_{D}^{*}\left(T\right)=\frac{R}{e^{\frac{B}{T}}-F}+O,$$

where *R*, *B*, *F*, *O* are constants determined at the calibration stage of the thermal imaging camera. For the above assumptions, Formula (23) can be written in the form:

(25) $$\begin{array}{ccc} U_{tot}^{*} & = & A_{D}F_{dD-Q}\cdot \tau_{Q}\varepsilon_{o}\tau_{a}\left(\frac{R}{e^{\frac{B}{T_{o}}}-F}+O\right)+\\ & & A_{D}F_{dD-Q}\cdot \tau_{Q}\left(1-\varepsilon_{o}\right)\tau_{a}\left(\frac{R}{e^{\frac{B}{T_{r}}}-F}+O\right)+\\ & & A_{D}F_{dD-Q}\cdot \tau_{Q}\left(1-\tau_{a}\right)\left(\frac{R}{e^{\frac{B}{T_{a}}}-F}+O\right)+\\ & & A_{D}F_{dD-V}\cdot \tau_{Q}\varepsilon_{V}\left(\frac{R}{e^{\frac{B}{T_{V}}}-F}+O\right)+\\ & & A_{D}F_{dD-H}\cdot \varepsilon_{H}\left(\frac{R}{e^{\frac{B}{T_{H}}}-F}+O\right). \end{array}$$

For known values of the constants *R*, *B*, *F*, and *O*, the temperature values of the observed object $T_{o}$ can be calculated for a given voltage value $U_{tot}^{*}$ by transforming Formula (25) to the form:

(26) $$T_{o}=\frac{B}{\ln\left[\frac{R}{\frac{U_{tot}^{*}-A_{D}F_{dD-Q}\cdot \tau_{Q}\left(1-\varepsilon_{o}\right)\tau_{a}U_{D}^{*}\left(T_{r}\right)-A_{D}F_{dD-Q}\cdot \tau_{Q}\left(1-\tau_{a}\right)U_{D}^{*}\left(T_{a}\right)-U_{V}\left(T_{V}\right)-U_{H}\left(T_{H}\right)}{A_{D}F_{dD-Q}\cdot \tau_{Q}\varepsilon_{o}\tau_{a}}-O}+F\right]}$$

where the designations $U_{V}\left(T_{V}\right)$ and $U_{H}\left(T_{H}\right)$ are defined by Formulas (17) and (22), respectively.

To increase the accuracy of temperature measurements based on the analysis of thermographic data, the model employs two blackbodies (BB). Using this BB set allows for correcting the unknown measured temperature of the object by registering the radiation from blackbodies with known temperatures. Both the blackbodies and the object are visible in the same thermographic image. When two blackbodies are used, the temperature correction is performed using the following formula:

(27) $$T_{o}^{*}=\frac{T_{BB_{2}}-T_{BB_{1}}}{{\bar{T}}_{o2}-{\bar{T}}_{o1}}\cdot \left(T_{o}-{\bar{T}}_{o1}\right)+T_{BB_{1}}$$

where ${\bar{T}}_{o1}$ and ${\bar{T}}_{o2}$ are the average temperature values calculated (Equation (28)) from the temperature values of each of the *N* pixels containing the reference blackbody image with temperatures $T_{BB_{1}}$ and $T_{BB_{2}}$, respectively, according to the formulas:

(28) $${\bar{T}}_{o1}=\frac{1}{N}\sum_{n=1}^{N}T_{o1n},\;{\bar{T}}_{o2}=\frac{1}{N}\sum_{n=1}^{N}T_{o2n}.$$

$T_{o1n}$ and $T_{o2n}$ temperature values are calculated using Formula (26).

In a thermal imaging camera, the emissivity value of the measured object, the emissivity value of the reference blackbodies and their temperature, and the temperature $T_{r}$ are known and entered into the algorithm by the camera operator. In determining the measured temperature correction, all of the aforementioned parameters are taken into account (Equation (26)). Consequently, the temperature measurement error is independent of the emissivity of the object when the latter exceeds 0.75. Conversely, for emissivities below this value, the determination of $T_{r}$ is subject to a significant error (in accordance with ISO 18434-1:2008 [28]). Therefore, the camera can be employed to measure the temperature of human skin, for which the emissivity is 0.98.

## 4. Laboratory Stand

### 4.1. Stand for Parameter Determination and Calibration

When measuring the temperature of an object according to Formula (26), the value of the bolometer voltage is determined using the approximating Function (24). Using the approximating function requires the values of its parameters *R*, *B*, *F*, and *O*. A special automated test stand was developed to determine the values of the parameters *R*, *B*, *F*, and *O*. The stand consists of the following elements:

- a set of blackbodies (placed on a linear motion drive),
- linear motion drive with a controller,
- climate chamber,
- computer with software for control and data recording.

**Figure 6:**
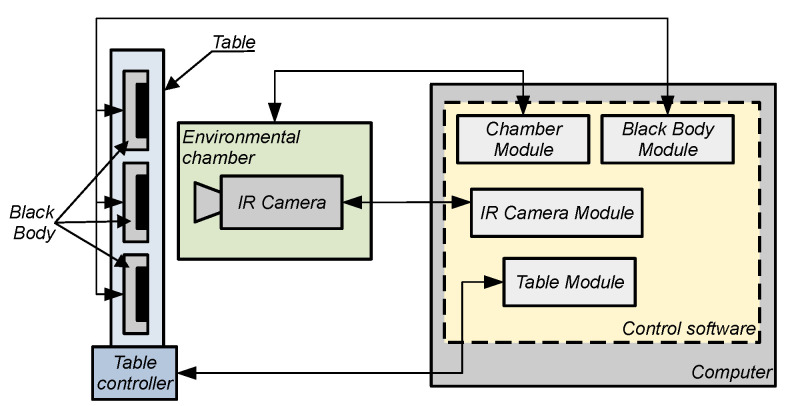
Block diagram of the station for determining the parameters and calibration of the radiometric thermal camera.

**Figure 7:**
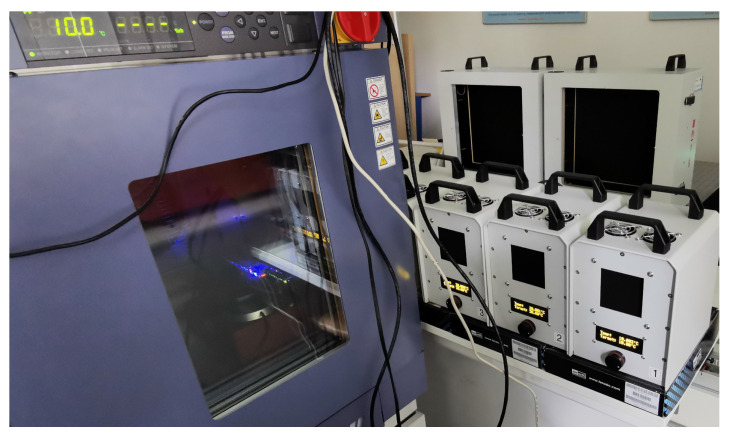
View of the workstation for determining the parameters and calibration of the radiometric thermal camera.

A block diagram of the station for data recording and determination of thermal camera parameters is shown in Figure 6, while Figure 7 presents a photo of the station during microbolometer thermal camera measurements.

The essential component of the thermal camera parameter determination bench is the control software. This software records thermographic data from the thermal camera via the Ethernet interface, allowing data transfer to a computer without additional hardware. Additionally, the computer is used to set blackbody parameters, control the linear drive for moving the blackbodies, and manage the climate chamber.

**Figure 8:**
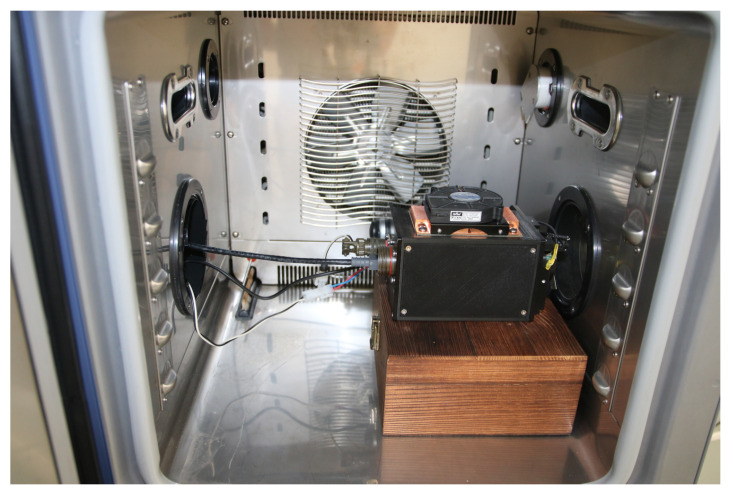
View of the thermal imaging camera inside the climate chamber.

One of the most important factors affecting the operation of the thermal imaging camera is the ambient temperature. In order to analyze the measured and determined parameters of the thermal imaging camera as a function of ambient temperature, the automated test stand was equipped with an SH-661 climate chamber from ESPEC (Japan). The basic parameters of the chamber are listed in Table 2, while Figure 8 shows a photo of the climate chamber with the microbolometer thermal imaging camera inside.

An essential component of the thermal imaging camera determination station is the blackbody. Its task is to generate infrared radiation that uniformly illuminates the detector array of the thermal imaging camera under test. The generated radiation flux depends on the blackbody’s temperature, which is why the blackbody has a high emissivity coefficient and very good temperature stability. Its selected parameters are listed in Table 3.

In order to automatically change the blackbody observed by the thermal imaging camera, the BB set was placed on a motorized linear stage. Adjustment of the stage’s position is carried out by a built-in controller driven by the main control software via an Ethernet communication interface.

### 4.2. Test Stand for Method Verification

In order to test the accuracy of remote temperature measurements using a thermal imaging camera with an implemented temperature determination method, the camera was tested under strictly controlled conditions. Accordingly, a special designed test stand was developed to evaluate the effectiveness of temperature measurement using the developed measurement system. This test stand consists of the following elements: a set of blackbodies with adjustable temperatures (forming the observed scene), a climate chamber, and a computer with software for control and data recording.

**Figure 9:**
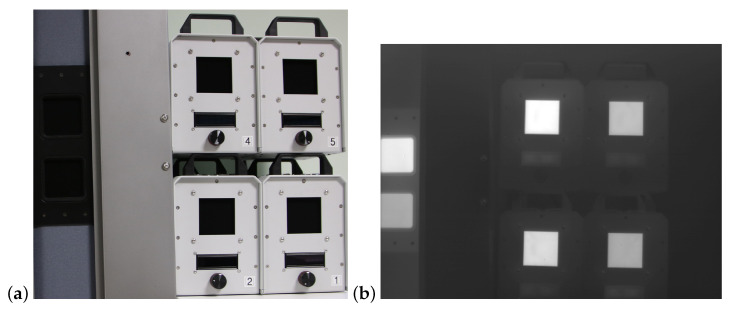
View of blackbodies at the test stand (**a**) and an example of a thermal image recorded at the stand for testing the accuracy of temperature measurement (**b**).

The blackbodies were placed in the field of view of the thermal imaging camera so that they formed the test measurement scene shown in Figure 9a (static arrangement of blackbodies). Software installed on the computer allows recording thermal images of the object and the observed blackbodies simultaniously. During the measurement, the temperature of the climate chamber and the temperature of the camera’s internal components were controlled. Figure 9b shows an example of a thermal image recorded by the thermal imaging camera under test.

## 5. Uncertainty Analysis of Temperature Measurements

One of the most important parameters of a measurement thermal camera is its specific spread of measured temperature values, i.e., the measurement uncertainty [29]. The uncertainty of temperature measurement with the developed thermal imaging camera can be determined from the BB temperature measurement. The absolute error of such a measurement is determined by the formula [30]:

(29) $$\Delta T=T_{D}-T_{BB},$$

where $T_{D}$—the temperature value measured by the thermal imaging camera, $T_{BB}$—the ”real” value of the temperature indicated by the blackbody, ΔT—the boundary uncertainty of the temperature measurement.

In the adapted method of temperature measurement, error measurement uncertainty is due to:

- statistical scatter of measurement results ($u_{T}$)—uncertainty type A (total scatter of the system),
- the maximum uncertainty of the standard temperature measurement ($u_{BB}$)—type B uncertainty.

Therefore, the standard uncertainty [29] can be described by the following formula:

(30) $$u=\sqrt{u_{T}^{2}+u_{BB}^{2}}.$$

The type A evaluation of uncertainty [29] is to determine the uncertainty based on statistical analysis of a series of *N* direct measurements (*N*—element sample) $\Delta T_{1},\;\Delta T_{2},\;\ldots \;\Delta T_{N}$. If the conditions provide the same accuracy of independent measurements, the random variable, which is the result of a single measurement, is subject to a normal distribution (Gaussian distribution). The arithmetic mean of *N* measurement results is taken as the measurement result:

(31) $$\bar{T}=\frac{1}{N}\sum_{i=1}^{N}T_{i},$$

which is considered a good estimate of the true value. In this case, the value of the standard uncertainty of the result of measurement of a quantity can be determined from the formula:

(32) $$u_{T}=\sqrt{\frac{1}{N(N-1)}\sum_{i=1}^{N}{\left(T_{i}-\bar{T}\right)}^{2}}.$$

A fundamental factor affecting the uncertainty of temperature measurement in the applied error analysis procedure is the temperature standard used (blackbody). For such a case, the type B evaluation of uncertainty [29] is related to the maximum uncertainty of the temperature measurement of the standard. If the value of the calibration uncertainty of the blackbody used is $\Delta T_{BB}$, then, assuming that the probability density function of this quantity is a uniform (rectangular) distribution, the standard uncertainty can be calculated from the formula:

(33) $$u_{BB}\left(x\right)=\frac{\Delta T_{BB}}{\sqrt{3}}.$$

From the above assumptions and from Formula (30), the standard uncertainty of temperature measurement with the developed thermal imaging camera is described by the formula:

(34) $$u=\sqrt{\frac{1}{N(N-1)}\sum_{i=1}^{N}{\left(T_{i}-\bar{T}\right)}^{2}+\frac{\Delta T_{BB}^{2}}{3}}.$$

The standard uncertainty *u* defines the interval from x−u to x+u in which the true value of the measured temperature is located. In order to be able to compare the results of temperature measurements obtained under different conditions, it is necessary to determine the expanded uncertainties *U* [29]. The expanded uncertainty is the increased value of the standard uncertainty so that in the interval x±U is the predominant number of results obtained. In the case of temperature measurement with a thermal imaging camera, it was assumed that the expanded uncertainty would be determined according to the formula:

(35) U=k·u,

where *k* is the coverage factor. In the adopted uncertainty evaluation procedure, it was assumed that the probability distribution of the temperature measurement result has an approximately normal distribution. Then, for the coverage factor k=2, the confidence level is 95% (it is evident that the measured temperature exhibits a Gaussian distribution, with an assumed interval of 2σ).

## 6. Measurement Results

As a part of this research work, a measurement system was developed with an implemented algorithm characterized by the following basic parameters:

- resolution: 640 × 480,
- size of a single detector: 17 µm,
- frame rate (number of images per second): 25 Hz,
- temperature measurement range: 28–42 °C,
- temperature measurement resolution: ≤0.08 °C.

**Figure 10:**
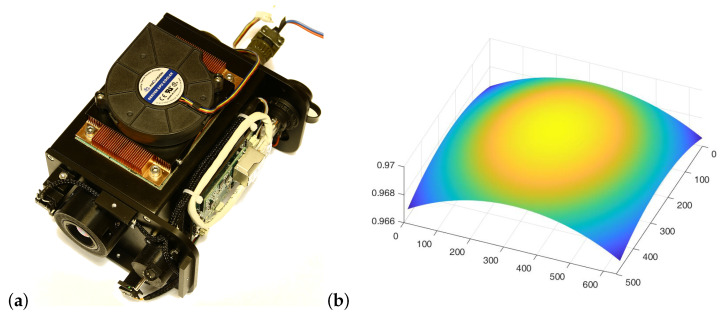
Microbolometer thermal camera with the thermal sensors (**a**) and values of the configuration factors $F_{dD-Q}$ between the surface of the detector array *D* and the surface *Q* (**b**).

The core of the system is a microbolometer thermal camera, developed at IOE MUT, equipped with additional temperature sensors to measure the actual temperature values $T_{a}$, $T_{H}$, and $T_{V}$. The temperature value $T_{r}$ is entered by the system operator. The configuration factors $F_{dD-Q}$, $F_{dD-V}$, and $F_{dD-H}$ were numerically determined for the entire measurement system. Figure 10 shows a view of the microbolometer camera and an example of the distribution of configuration factors $F_{dD-Q}$.

In order to determine the values of *R*, *B*, *F*, and *O* parameters on the calibration stand, the so-called radiometric calibration was carried out. Radiometric calibration consisted of measuring the detector’s voltage $U_{tot}$ for different values of object temperature $T_{o}$, housing temperature $T_{H}$, and lens temperature $T_{V}$. The measured object was a set of blackbodies, and the camera was placed in a climate chamber allowing controlled setting of $T_{H}$ and $T_{V}$ temperatures. Tests were conducted at eight distinct temperature values $T_{a}$ within the climate chamber. For each temperature value $T_{a}$, the temperatures $T_{H}$ and $T_{V}$ were measured using the internal sensors in the camera, and eight sets of temperature values $T_{o}$ were recorded. Each set of $T_{o}$ temperatures comprised data obtained from an area of 35×35 pixels at a specific blackbody temperature setting. The measurements yielded K=200 measurement points composed of voltage values $U_{k}$ and corresponding temperatures $T_{ok}$, $T_{Hk}$, $T_{Vk}$. This means that 200 measurements are taken for one temperature $T_{a}$ and one pixel (from the area 35×35). In this case, the primary focus is on preserving the general shape of the curve (approximating function) that best fits the measured data points. Least-squares optimization was used for this task. The calculations necessary to determine the parameters *R*, *B*, *F*, and *O* by the method of least squares were performed using digital data processing methods. Based on this, the following values of the *R*, *B*, *F*, and *O* parameters were obtained:

$$R=3.297336\times 10^{6},$$

$$B=1.233238\times 10^{3},$$

$$F=1.125584\times 10^{2},$$

$$O=1.071801\times 10^{5}.$$

The determined parameters yielded a function Fit (26), characterized by an average relative error of less than 0.13% and a maximum relative error of less than 0.3%.

**Figure 11:**
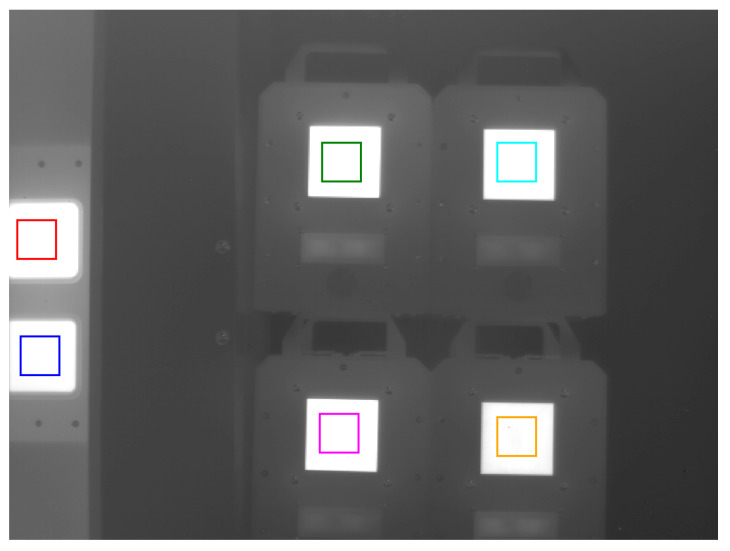
View of the measurement situation with four test blackbodies.

The developed thermal imaging camera, featuring an algorithm for determining temperature that accounts for changes in ambient temperature and corrects measurements based on blackbodies’s known radiation, was tested during a measurement session on the test bench. During the tests, a scene was recorded with objects—blackbodies, whose temperatures were measured in the presence of additional blackbodies acting as reference sources. The measurement situations are shown in Figure 11.

The measurement locations (Figure 11) were marked with rectangles colored in green, cyan, magenta, and orange for the test blackbodies. Red and blue (Figure 11) were used to mark locations as reference sources for temperatures set by the camera (two blackbodies used to correct the measured temperature according to Formula (27)). The temperature was measured at four measurement points. The temperatures of the test blackbodies are summarized in Table 4. In order to increase the reliability of the testing of the temperature measurement method, different temperature values were set on the test bodies rather than during calibration.

**Figure 12:**
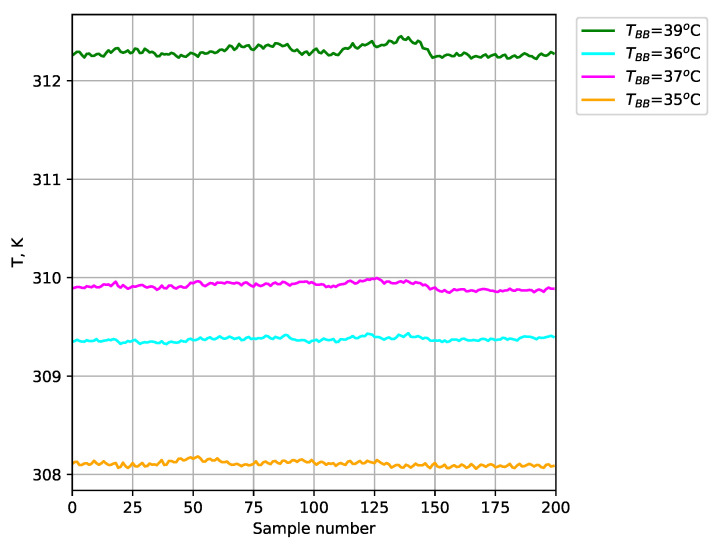
Plot of the measured temperature for the test blackbodies.

**Figure 13:**
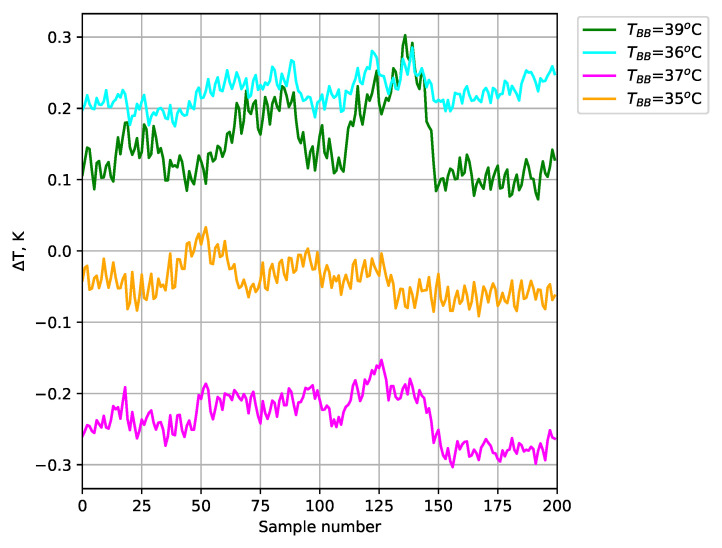
Absolute error of temperature measurement for test blackbodies.

**Figure 14:**
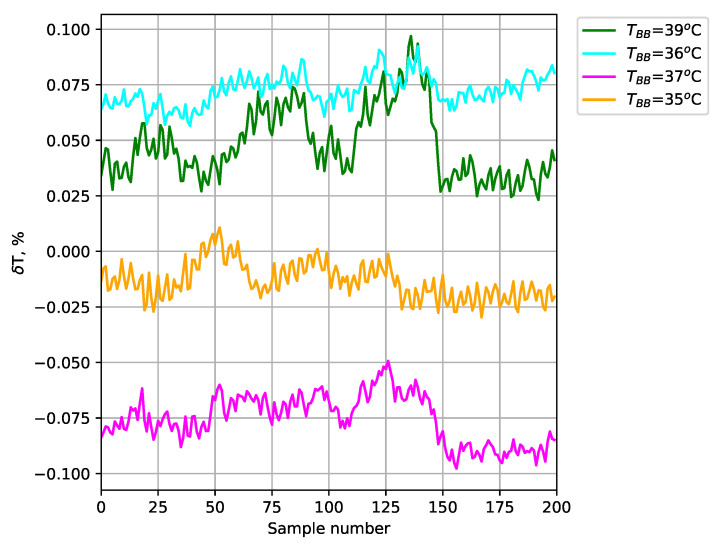
Relative error of temperature measurement for test blackbodies.

**Figure 15:**
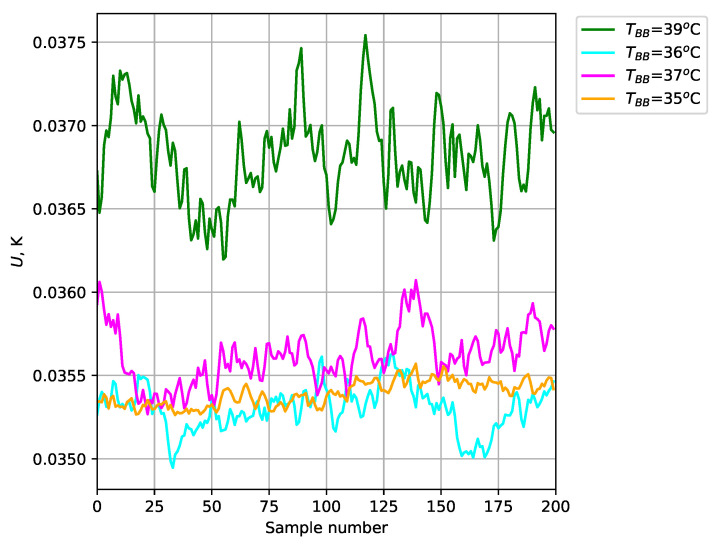
Expanded uncertainty with 95% confidence level for test blackbodies.

The obtained temperature results for the test blackbodies are shown in Figure 12 (average of a 35×35 area). The absolute error (Figure 13), relative error (Figure 14), and expanded uncertainty with a 95% confidence level (Figure 15) were calculated for the temperature measured at each point.

The use of blackbodies and a climate chamber allowed for precise control of the observed scene and environmental conditions, including ambient temperature and humidity in particular. The results obtained confirmed the high accuracy of temperature measurements with the developed thermal imaging camera. The maximum absolute error for the measured reference bodies did not exceed 0.32 K. Small values of expanded uncertainty with a confidence level of 95% were also obtained for the reference blackbodies (less than ±0.038 K).

## 7. Conclusions

**Figure 16:**
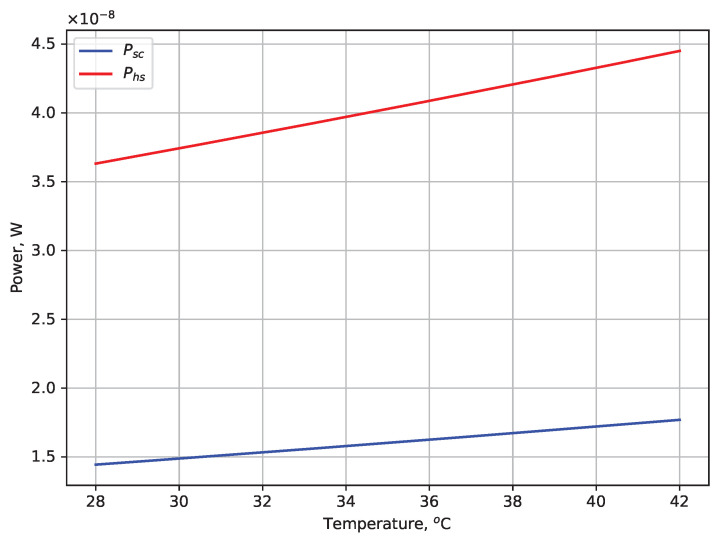
Radiant power of the scene ($P_{sc}$, blue) and the summed power of all other interfering signals ($P_{sh}$, red) as a function of temperature.

The novel method of calibrating a thermal imaging camera presented in this article makes it possible to achieve high accuracy in measuring the temperature of the observed object. This method was developed based on a radiation model of temperature measurement, assuming that the detector’s voltage can be separated into a useful signal, which reflects the power of incident radiation from the observed scene, and an undesired interfering signal from other sources of radiation. The analysis of the adopted model shows that for a scene and camera enclosure temperatures close to 30 °C, the power of that undesired radiation is about 2.5 times greater than that of radiation incident from the scene (Figure 16).

The developed model takes into account several factors that significantly affect the accuracy of temperature measurement. In particular, the distribution of configuration coefficients for each detector in the array, as well as changes in temperature of system components such as the housing and lens. Additionally, to enhance measurement accuracy, two reference blackbodies with known temperatures were placed within the field of view of the thermal camera as reference sources. The effectiveness of the calibration method was confirmed by applying it to the developed measurement system using a specially constructed thermal imaging camera. Temperature measurements of observed objects were then conducted with this system under strictly controlled conditions. The results obtained allow us to conclude that the developed calibration method enables the practical implementation of a thermal imaging system capable of highly accurate remote temperature measurement. This system can be effectively utilized in high-accuracy demanding applications such as veterinary medicine, the food industry, and human body temperature screening.

## Figures and Tables

**Table 1:** Parameters of the lens used in the thermal imaging camera module.

Parameter	Value
Spectral range	LWIR 8–14 µm
Focal length	19 mm
Angles of field of view for 640 × 480@17 µm	32° × 24°
F-number (aperture number)	f/1.0
Transmission	>94%
Operating temperature range	−40 °C to +80 °C

**Table 2:** Basic parameters of the SH-661 climate chamber by ESPEC (Japan).

Parameter	Value
Temperature range	−60 °C – +150 ° C
Humidity control	30 to 95% rh
Internal dimensions	400 × 400 × 400 mm
Communication interface with computer	RS485 serial port

**Table 3:** Basic parameters of BB developed and made at IOE MUT (Poland).

Parameter	Value
Size of blackbody surface	62 mm × 62 mm
Absolute temperature stabilization range	10 °C – 60 ° C
	(for ambient temperature of 20 °C)
Average directional emissivity at an angle of 20°	0.985
Average directional emissivity at an angle of 60°	0.973
Average hemispheric emissivity	0.944
Temperature setting accuracy	0.01 ° C
Total temperature uncertainty	0.03 °C
Stability	0.01 °C

**Table 4:** Temperatures of test blackbodies (Figure 11).

$T_{\mathrm{BB}}$, °C (K)	Color
39.0 (312.15)	green
36.0 (309.15)	cyan
37.0 (310.15)	magenta
35.0 (308.15)	orange

## Data Availability

Data used in the study is available upon request.
